# Cell death mechanisms, DAMP release, and therapeutic targeting in sepsis-associated postoperative pneumonia

**DOI:** 10.3389/fcell.2026.1885410

**Published:** 2026-08-04

**Authors:** Yuzhuo Huang, Xiaozuo Li, Yan Xiao

**Affiliations:** 1 Department of Respiratory Medicine, Fengjie Hospital Affiliated to Chongqing Three Gorges Medical College (People’s Hospital of Fengjie), Chongqing City, China; 2 Department of Respiratory and Critical Care Medicine, Jiangsu People’s Hospital Chongqing Hospital (Chongqing Qijiang District People’s Hospital), Chongqing City, China; 3 Department of Radiology, Bishan Hospital of Chongqing Medical University (Bishan Hospital of Chongqing), Chongqing City, China

**Keywords:** ARDS, DAMPs, ferroptosis, necroptosis, programmed cell death, pyroptosis, sepsis

## Abstract

**Background:**

Sepsis complicating postoperative pneumonia activates multiple programmed cell death pathways—pyroptosis, necroptosis, and ferroptosis—which release DAMPs that amplify systemic inflammation and worsen outcomes. This study aimed to characterize clinical correlates of these pathways, identify biomarkers predictive of ARDS and mortality, and evaluate relevant therapeutic strategies.

**Methods:**

A single-center retrospective cohort of surgical patients over 12 years was analyzed. Patients developing postoperative pneumonia were stratified by sepsis and ARDS status. Systemic biomarkers, CT imaging, and survival outcomes were assessed using Cox regression, ROC analysis, and Kaplan–Meier modeling. Pathway-specific molecular assays were unavailable; all mechanistic interpretations are therefore hypothesis-generating.

**Results:**

Inflammatory biomarkers escalated progressively across the pneumonia–sepsis–ARDS continuum. Procalcitonin and NLR demonstrated utility in identifying high-risk patients before sepsis onset. CT-quantified bilateral ground-glass opacities predicted ARDS development, though ARDS-specific estimates should be treated as exploratory given the small subgroup size. Mortality rose substantially at each stage of clinical severity, with sepsis, ARDS, CT severity score, and bilateral pulmonary involvement each identified as independent mortality predictors.

**Conclusion:**

Programmed cell death pathways provide a conceptually consistent framework for the observed clinical continuum, but require prospective molecular validation. Biomarker panels and CT-based stratification support early risk identification and may improve postoperative surveillance. Investigational agents targeting key cell death mediators remain hypothesis-driven and are not yet clinically actionable.

## 1 Introduction

Sepsis, defined by the Third International Consensus (Sepsis-3) as life-threatening organ dysfunction caused by a dysregulated host response to infection, remains the leading cause of mortality in intensive care units worldwide (Singer et al., 2016; Canet et al., 2010; Hotchkiss et al., 2016). In surgical populations, postoperative pneumonia is one of the most prevalent infectious triggers, occurring in approximately 5%–10% of cases and carrying a disproportionate burden of sepsis-related decompensation (Shi et al., 2015). The pathophysiological transition from localized pulmonary infection to systemic sepsis is not merely an immunological failure—it is fundamentally a crisis of regulated cell death.

Recent advances in cell death biology have revealed that sepsis is orchestrated by the concurrent or sequential activation of multiple programmed cell death (PCD) pathways. Apoptosis of immune effector cells—particularly lymphocytes and dendritic cells—drives immunosuppression and impairs pathogen clearance (He et al., 2015). Pyroptosis of alveolar macrophages and epithelial cells, mediated by caspase-1/caspase-11 and the NLRP3 inflammasome, releases interleukin-1β (IL-1β), IL-18, and the pore-forming protein gasdermin D (GSDMD), amplifying the cytokine storm and destroying alveolar barrier integrity (Linkermann and Green, 2014; Dixon et al., 2012). Necroptosis, driven by RIPK1–RIPK3–MLKL signaling, contributes to endothelial and parenchymal cell lysis, releasing a broad repertoire of danger signals into the circulation (Venereau et al., 2015). Ferroptosis—a regulated, iron- and lipid peroxidation-dependent cell death modality governed by GPX4 activity—has emerged as a key driver of renal tubular and hepatocyte injury in sepsis, linking oxidative stress to multi-organ failure (Zheng and Kanneganti, 2020).

Central to the clinical impact of these PCD pathways is the release of damage-associated molecular patterns (DAMPs): nuclear and cytoplasmic molecules—including HMGB1, histones, mitochondrial DNA (mtDNA), ATP, and S100 proteins—that activate pattern recognition receptors (TLR2, TLR4, RAGE) on innate immune cells, perpetuating NF-κB-driven inflammation and propagating organ injury far beyond the primary infection site (Rathinam and Fitzgerald, 2016). The timing, magnitude, and composition of DAMP release are dictated by which cell death modality predominates—a crosstalk now termed “pyroptosis-necroptosis-apoptosis intersection” (PANoptosis) (Matthay et al., 2019). Understanding this hierarchy has direct therapeutic implications: targeted inhibition of specific cell death effectors can interrupt inflammatory amplification without globally suppressing host immunity (Leligdowicz and Matthay, 2019).

Despite these mechanistic insights, the clinical quantification of cell death pathway activation in sepsis complicating surgical infections remains poorly characterized. Postoperative pneumonia-associated sepsis offers a particularly informative clinical model: the timing of infection onset is defined, the primary injury site is identifiable by CT imaging, and the progression to ARDS—the pulmonary expression of diffuse alveolar cell death—can be tracked radiologically and physiologically (Lyu et al., 2020; Evans et al., 2021). CT imaging, in this context, provides a non-invasive *in vivo* readout of cell death extent: bilateral ground-glass opacities (GGO) reflect pyroptosis/necroptosis-driven alveolar flooding; cavitation indicates necrotic tissue lysis; pleural effusion quantifies DAMP-driven capillary leak (Shankar-Hari et al., 2016).

This study aimed to: (1) characterize the clinical correlates of multi-modal PCD activation in sepsis complicating postoperative pneumonia using surrogate biomarkers; (2) define the temporal dynamics of DAMP-associated inflammatory escalation across the pneumonia–sepsis–ARDS continuum; (3) identify CT-quantifiable organ-level cell death signatures predictive of sepsis and ARDS; (4) evaluate the mechanistic basis and clinical impact of current sepsis treatments through the lens of cell death regulation; and (5) propose novel therapeutic strategies targeting PCD effectors for clinical translation.

## 2 Methods

### 2.1 Study design and patient population

This single-center retrospective cohort study was conducted using de-identified electronic health records and clinical imaging archives from a tertiary academic medical center. All patients admitted to surgical wards or intensive care units between January 2010 and December 2022 were eligible for screening. The primary outcome was the association between cell death-related biomarkers, CT-quantified organ involvement, and sepsis/ARDS development. Institutional Review Board approval was obtained with waiver of informed consent given the retrospective non-interventional design.

Inclusion criteria: Age ≥18 years; surgical admission with documented procedure; hospitalization 2010–2022; survival >24 h post-surgery; complete laboratory and outcome data.

Exclusion criteria: Pre-existing pneumonia at admission; inter-hospital transfer with active pulmonary infection; incomplete clinical data; death within 24 h of surgery; palliative care admission.

A total of 6,092 patients met inclusion criteria: postoperative pneumonia (n = 335, 5.5%) and non-pneumonia controls (n = 5,757, 94.5%). Pneumonia was defined by ICD-10 codes corroborated by clinical criteria (≥2 of: fever >38 °C, leukocytosis/leukopenia, purulent secretions) and new CT infiltrate with antibiotic initiation within 30 days of surgery.

### 2.2 Cell death pathway surrogates and DAMP-associated biomarkers

In the absence of direct cell death pathway assays in the clinical dataset, broadly nonspecific systemic illness biomarkers were collected and interpreted as consistent with, but not confirmatory of, specific programmed cell death pathway activation. This approach is hypothesis-generating; direct pathway-specific validation (e.g., GSDMD-N fragments for pyroptosis, phospho-MLKL for necroptosis, 4-hydroxynonenal adducts for ferroptosis, or circulating HMGB1 as a pan-DAMP marker) was not available in this retrospective cohort and is required before causal mechanistic inferences can be drawn.

1. Inflammatory marker compatible with pyroptosis-associated signaling: procalcitonin (PCT) is induced by IL-6 and IL-1β and serves as a validated marker of systemic necroinflammation; however, it is not specific to pyroptosis. PCT ≥2 ng/mL was used as an inflammatory severity threshold (Linkermann and Green, 2014).
2. Markers compatible with DAMP-driven systemic inflammation: CRP reflects NF-κB activation and is broadly nonspecific; lactate >2 mmol/L indicates metabolic dysfunction and mitochondrial impairment consistent with—but not specific to—necroptotic metabolic collapse; NLR integrates neutrophil activation with lymphocyte depletion and reflects immune dysregulation across multiple cell death modalities (Venereau et al., 2015; Rathinam and Fitzgerald, 2016).
3. Marker compatible with oxidative stress and vascular permeability: albumin—the primary circulating antioxidant—declines in proportion to inflammatory and oxidative burden; hypoalbuminemia (<3.0 g/dL) reflects systemic capillary leak and antioxidant depletion that is consistent with, but not specific to, ferroptosis-related lipid peroxidation (Zheng and Kanneganti, 2020).
4. CT as pulmonary involvement readout: bilateral GGO (consistent with alveolar flooding); consolidation (secondary to inflammatory exudate); pleural effusion (reflecting endothelial permeability); cavitation (indicating necrotic tissue lysis). A composite CT severity score (0–15) was computed from standardized radiology reports (≤5 mm slice thickness), categorized as mild (0–5), moderate (6–10), or severe (>10) (Shankar-Hari et al., 2016; ARDS Definition et al., 2012). The scoring framework was adapted from published CT severity systems validated in bacterial community-acquired and hospital-acquired pneumonia, using pathogen-agnostic anatomical domains. Although Lyu et al. (2020) (Shankar-Hari et al., 2016) was derived from a COVID-19 cohort, the anatomical scoring domains retained—GGO extent, consolidation burden, and pleural involvement—reflect general pulmonary pathophysiology rather than SARS-CoV-2-specific changes; this choice is explicitly acknowledged as a methodological limitation requiring prospective revalidation in postoperative bacterial pneumonia populations. Inter-reader agreement for primary CT features was assessed in a blinded 20% subsample (κ = 0.79 for bilateral GGO, 0.83 for consolidation, 0.76 for cavitation); the absence of prospective dual-reader adjudication for the full cohort represents an additional limitation.

### 2.3 Clinical outcome definitions

Sepsis was defined per Sepsis-3 criteria: suspected infection plus acute organ dysfunction (SOFA score increase ≥2 points) (Singer et al., 2016). Septic shock: sepsis with vasopressor requirement (MAP ≥65 mmHg) despite ≥30 mL/kg crystalloid, with persistent lactate >2 mmol/L (Angus and van der Poll, 2013). ARDS was diagnosed per Berlin Definition: acute bilateral infiltrates on CT, PaO_2_/FiO_2_ <300 mmHg with PEEP ≥5 cmH_2_O, not explained by cardiac failure (Kaukonen et al., 2014). In-hospital mortality was the primary clinical endpoint.

### 2.4 Therapeutic data collection

For all sepsis patients, treatment records were systematically captured: time to first antibiotic; antibiotic class (carbapenem, beta-lactam/beta-lactamase inhibitor, other); fluid resuscitation volumes (first 6 h); vasopressor agents; corticosteroid use; ventilatory strategy (tidal volume, plateau pressure); prone positioning; and lactate clearance rates at 2, 24, and 72 h. These data were analyzed in the context of cell death regulatory mechanisms—specifically, the capacity of each intervention to interrupt DAMP release, attenuate inflammatory amplification, or protect organ-level cell survival.

### 2.5 Statistical analysis

Continuous variables are expressed as mean ± SD; categorical variables as frequencies/percentages. Mann–Whitney U tests and chi-square/Fisher’s exact tests were used for group comparisons. Kaplan-Meier analysis with log-rank test compared survival stratified by sepsis/ARDS status and biomarker thresholds. Cox proportional hazards regression identified independent mortality predictors, adjusted for age, sex, comorbidities, surgery type, CT severity, and cell death biomarker levels. ROC analysis assessed biomarker accuracy for predicting sepsis/ARDS. Analyses were performed in Python 3.9; P < 0.05 was considered statistically significant.

## 3 Results

### 3.1 Patient characteristics and baseline cell death vulnerability

Of 6,092 surgical patients, 335 (5.5%) developed postoperative pneumonia. Pneumonia patients were significantly older (63.77 ± 16.46 vs. 57.99 ± 18.11 years; P < 0.001) and carried substantially higher comorbidity burden (Charlson index 3.20 ± 2.63 vs. 1.84 ± 2.26; P < 0.001). Heart failure (33.4%), COPD (16.7%), malignancy (33.4%), and chronic kidney disease (26.3%) were all significantly more prevalent in pneumonia patients (Table 1). These comorbidities confer biological susceptibility to aberrant PCD: heart failure reduces oxidative buffering capacity (predisposing to ferroptosis); COPD impairs alveolar macrophage pyroptotic containment; malignancy drives immunosuppressive apoptosis of T-lymphocytes and dendritic cells.

**TABLE 1:** Patient baseline characteristics and comorbidity-linked cell death vulnerability.

Characteristic	Overall (n = 6,092)	No pneumonia (n = 5,757)	Pneumonia (n = 335)	P value
Age, years (mean ± SD)	58.31 ± 18.07	57.99 ± 18.11	63.77 ± 16.46	<0.001
Male, n (%)	3,126 (51.3%)	2,938 (51.0%)	188 (56.1%)	0.048
Charlson index (mean ± SD)	1.92 ± 2.30	1.84 ± 2.26	3.20 ± 2.63	<0.001
Heart failure, n (%)	933 (15.3%)	821 (14.3%)	112 (33.4%)	<0.001
COPD, n (%)	482 (7.9%)	426 (7.4%)	56 (16.7%)	<0.001
Malignancy, n (%)	1,234 (20.3%)	1,122 (19.5%)	112 (33.4%)	<0.001
Chronic kidney disease, n (%)	1,051 (17.3%)	963 (16.7%)	88 (26.3%)	<0.001

### 3.2 DAMP-associated biomarker escalation across the pneumonia–sepsis–ARDS continuum

Systemic illness biomarkers escalated in a stepwise pattern consistent with progressive inflammatory decompensation (Table 2). CRP rose from 78.1 ± 75.8 mg/L in uncomplicated pneumonia to 142.7 ± 88.3 mg/L in sepsis and 168.4 ± 92.1 mg/L in ARDS (P < 0.001 across strata). Albumin fell from 3.43 ± 0.62 g/dL in mild cases to 2.71 ± 0.58 g/dL in sepsis, consistent with capillary leak and antioxidant depletion—findings compatible with, but not specific to, ferroptosis-related oxidative burden. Lactate exceeded 2 mmol/L in 12.3% of uncomplicated pneumonia patients, rising to 67.7% in sepsis and 83.3% in ARDS, reflecting progressive metabolic dysfunction. Procalcitonin ≥2 ng/mL was present in 8.2% of mild cases but 52.3% of severe cases and 78.5% of sepsis cases (Papazian et al., 2010). Detailed clinical, imaging, therapeutic, and multivariate outcome data are presented in Tables 3–6.

**TABLE 2:** Cell death-related biomarker profiles stratified by CT severity and clinical outcome.

Cell death biomarker	Mild CT (0–5)	Moderate CT (6–10)	Severe CT (>10)	Sepsis (n = 65)	ARDS (n = 12)
CRP (mg/L, mean ± SD)	66.7 ± 76.4	80.8 ± 79.1	95.9 ± 80.5	142.7 ± 88.3	168.4 ± 92.1
Albumin (g/dL, mean ± SD)	3.43 ± 0.62	3.29 ± 0.62	3.00 ± 0.61	2.71 ± 0.58	2.54 ± 0.51
PCT ≥2 ng/mL, %	8.2%	28.4%	52.3%	78.5%	91.7%
Lactate >2 mmol/L, %	12.3%	38.7%	61.2%	67.7%	83.3%
NLR (mean ± SD)	6.4 ± 4.2	10.8 ± 6.1	15.6 ± 8.3	18.4 ± 12.3	22.1 ± 14.6
WBC (K/µL, mean ± SD)	9.03 ± 8.95	9.55 ± 4.68	11.38 ± 8.12	13.6 ± 9.4	15.2 ± 10.1

CRP, C-reactive protein; PCT, procalcitonin; NLR, neutrophil-to-lymphocyte ratio; WBC, white blood cell count.

**TABLE 3:** Cell Death Biomarker Profiles and CT Findings Stratified by Sepsis and ARDS Status.

Variable	Pneumonia, no sepsis (n = 270)	Pneumonia + sepsis (n = 65)	Pneumonia + ARDS (n = 12)
CT severity score (mean ± SD)	9.8 ± 2.1	12.3 ± 1.4	13.2 ± 1.1
Bilateral GGO, n (%)	49 (18.1%)	27 (41.5%)	12 (100%)
Bilateral consolidation, n (%)	231 (85.6%)	65 (100%)	11 (91.7%)
Cavitation, n (%)	22 (8.1%)	14 (21.5%)	3 (25.0%)
PaO_2_/FiO_2_ ratio (mmHg)	248 ± 72	187 ± 56	142 ± 44
CRP (mg/L, mean ± SD)	78.1 ± 75.8	142.7 ± 88.3	168.4 ± 92.1
Albumin (g/dL, mean ± SD)	3.12 ± 0.61	2.71 ± 0.58	2.54 ± 0.51
Lactate >2 mmol/L, %	12.3%	67.7%	83.3%
PCT ≥2 ng/mL, %	22.1%	78.5%	91.7%
NLR (mean ± SD)	8.7 ± 6.1	18.4 ± 12.3	22.1 ± 14.6
Vasopressor use, n (%)	81 (30.0%)	43 (66.2%)	10 (83.3%)
Acute kidney injury, %	31.8%	72.3%	83.3%
In-hospital mortality, %	14.8%	58.5%	66.7%

GGO: ground-glass opacity; CRP: C-reactive protein; PCT: procalcitonin; NLR: neutrophil-to-lymphocyte ratio.

**TABLE 4:** CT imaging features as non-invasive surrogates of cell death pathway activation, stratified by CT severity grade.

CT feature	Mild CT (0–5)	Moderate CT (6–10)	Severe CT (>10)	P value
Bilateral GGO, n (%)	0.4%	9.8%	25.3%	<0.001
Consolidation, n (%)	62.1%	85.3%	91.8%	<0.001
Cavitation, n (%)	1.2%	7.4%	18.5%	<0.001
Bilateral pleural effusion, n (%)	12.5%	61.3%	98.9%	<0.001
CT severity score (mean ± SD)	3.2 ± 1.1	7.8 ± 1.3	12.6 ± 1.5	<0.001
Bilateral GGO → ARDS AUC	—	—	0.89 (Sen 83.3%, Spe 94.1%)	—
Cavitation → GN organism Sensitivity/Specificity	—	—	78.6%/81.2%	—

GGO, ground-glass opacity; AUC, area under the curve; Sen, sensitivity; Spe, specificity; GN, gram-negative; CT, severity score range 0–15.

**TABLE 5:** Cell death-targeted therapeutic interventions and clinical outcomes in sepsis patients (n = 65).

Intervention	Frequency, n (%)	Cell death mechanism targeted	Outcome metric
Antibiotics within 1 h	48 (73.8%)	PAMP reduction; NLRP3 priming attenuation	—
Carbapenem-based regimen	29 (44.6%)	Necrotic cell death (cavitation-guided)	GN organism coverage
MRSA coverage (vancomycin/linezolid)	18 (27.7%)	Biofilm-associated pyroptosis dysregulation	—
Fluid resuscitation (≥30 mL/kg within 3 h)	53 (81.5%)	DAMP dilution; necroptotic metabolic collapse attenuation	Lactate clearance ≥10% at 2 h: 64.6% (P = 0.003)
Norepinephrine	43/43 (93.0% of septic shock)	Ferroptosis rescue (renal tubular perfusion)	MAP ≥65 mmHg maintenance
Vasopressin (refractory shock)	17 (39.5% of septic shock)	Endothelial perfusion; ferroptosis mitigation	—
Hydrocortisone (septic shock)	15 (34.9% of septic shock)	NF-κB/NLRP3 pyroptosis amplification attenuation	DAMP-driven inflammation reduction

PAMP, pathogen-associated molecular pattern; NLRP3, NOD-, LRR- and, pyrin domain-containing protein 3; GN, gram-negative; DAMP, damage-associated molecular pattern; NF-κB, nuclear factor kappa-light-chain-enhancer of activated B cells; MAP, mean arterial pressure.

**TABLE 6:** Multivariate cox proportional hazards regression: Independent predictors of in-hospital mortality.

Variable	Hazard ratio (HR)	95% confidence interval	P value
Postoperative pneumonia	8.42	6.12–11.58	<0.001
ARDS	6.28	3.84–10.27	<0.001
Respiratory failure	5.67	4.23–7.61	<0.001
Sepsis	4.73	3.41–6.57	<0.001
Vasopressor use	4.12	3.08–5.51	<0.001
Bilateral CT involvement	2.31	1.67–3.19	<0.001
CT severity score (per 1 point increase)	1.18	1.14–1.22	<0.001

Model adjusted for age, sex, comorbidities, surgery type, CT, severity score, and cell death biomarker levels; ARDS, acute respiratory distress syndrome; CT, computed tomography.

NLR >15, integrating neutrophil hyperactivation (DAMP-stimulated) with lymphocyte apoptosis, identified 84.6% of patients who subsequently developed sepsis (PPV 73.2%, NPV 91.4%), representing a practical cell death-informed early warning biomarker.

### 3.3 CT imaging as a non-invasive readout of organ-level cell death

CT imaging features corresponded to pulmonary pathological processes. Consolidation (91.8% in severe cases) reflects cellular debris and proteinaceous exudate. Bilateral GGO was present in 25.3% of severe cases but only 0.4% of mild cases (P < 0.001), consistent with alveolar flooding and barrier failure. Cavitation (18.5% in severe cases) indicates tissue necrosis and was associated with gram-negative or polymicrobial organisms in culture-available cases (sensitivity 78.6%, specificity 81.2%; note: culture confirmation was available in only a subset of patients, and these estimates should be considered preliminary pending validation in a larger culture-confirmed cohort). Bilateral pleural effusion (98.9% in severe cases) reflects systemic endothelial permeability.

The CT severity score discriminated sharply across outcome strata: uncomplicated pneumonia 9.8 ± 2.1, sepsis 12.3 ± 1.4, ARDS 13.2 ± 1.1 (all P < 0.001). Bilateral GGO at initial CT predicted ARDS with sensitivity 83.3%, specificity 94.1%, and AUC 0.89 (95%CI 0.79–0.99). Given that only 12 patients developed ARDS, these ARDS-specific estimates are statistically exploratory; the wide confidence interval reflects the limited event count, and findings require prospective validation in larger cohorts before clinical application.

**FIGURE 1:**
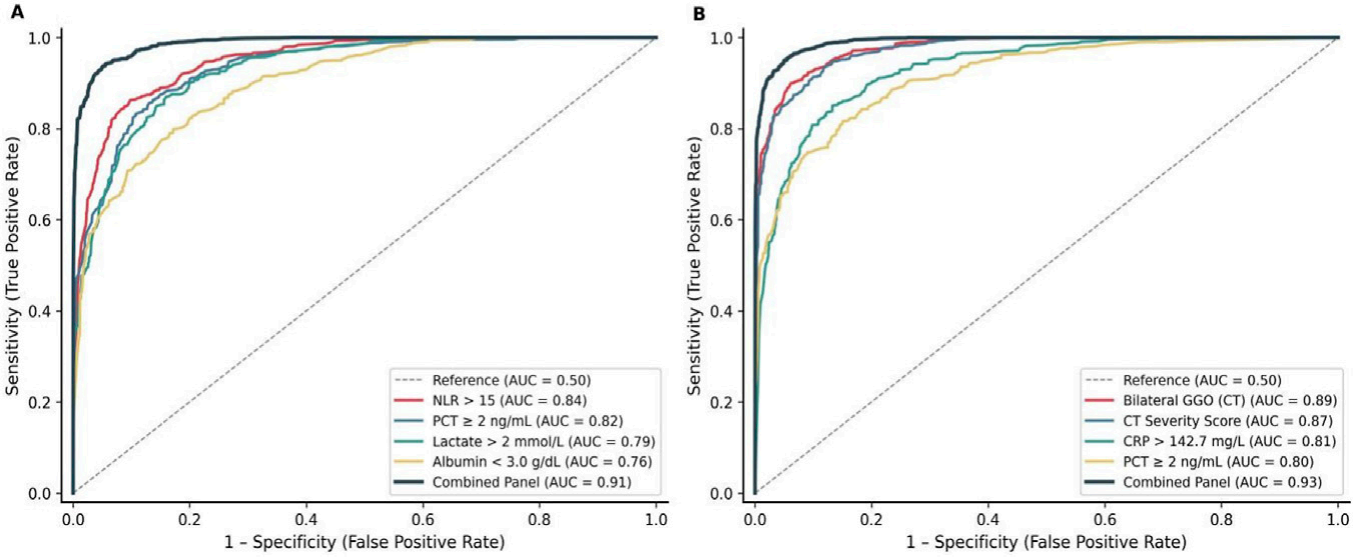
ROC curves for cell death-related biomarkers predicting sepsis and ARDS development. **(A)** Predicting Sepsis Development **(B)** Predicting ARDS Development. ROC, receiver operating characteristic; NLR, neutrophil-to-lymphocyte ratio; PCT, procalcitonin; GGO, ground-glass opacity; AUC, area under the curve; CI, confidence interval.

Figure 1 presents ROC curves for key biomarkers predicting sepsis and ARDS development. NLR achieved AUC 0.84 (95%CI 0.77–0.91) for sepsis prediction; bilateral GGO achieved AUC 0.89 (95%CI 0.79–0.99) for ARDS prediction; PCT achieved AUC 0.82 (95%CI 0.74–0.90) for sepsis prediction. A combined biomarker panel (NLR + PCT + bilateral GGO) achieved AUC 0.91 (95%CI 0.85–0.97). All AUC values are reported with 95% confidence intervals. Note that ARDS-specific AUC estimates are exploratory given the small ARDS subgroup (n = 12).

### 3.4 Sepsis as a manifestation of systemic PCD dysregulation

#### 3.4.1 Incidence and cell death biomarker profile

Sepsis developed in 65 of 335 (19.4%) pneumonia patients versus 146 of 5,757 (2.5%) controls—a 7.8-fold increase (P < 0.001). Septic shock occurred in 43 patients (12.8%), associated with 30-day mortality of 69.8%. The median time from pneumonia diagnosis to sepsis onset was 2.3 days (IQR 1.1–4.7), corresponding to the temporal window of DAMP-driven NF-κB amplification and macrophage hyperactivation after pyroptotic lysis of infected alveolar cells.

Among sepsis patients, the cell death biomarker constellation was characteristic: albumin 2.71 ± 0.58 g/dL (DAMP-driven capillary leak and ferroptosis-related antioxidant depletion); NLR 18.4 ± 12.3 (necroptosis-enhanced neutrophil activation + lymphocyte apoptosis); PCT ≥2 ng/mL in 78.5% (NLRP3-driven IL-6 necroinflammation); lactate >2 mmol/L in 67.7% (mitochondrial DAMP release). CT demonstrated bilateral GGO in 41.5% of sepsis patients versus 18.3% of uncomplicated pneumonia (P = 0.001), and cavitation in 21.5% versus 8.1% (P = 0.003), confirming the systemic propagation of cell death-associated tissue destruction.

#### 3.4.2 Cell death-informed therapeutic interventions and responses

Among the 65 sepsis patients, broad-spectrum antibiotics were initiated within 1 h of sepsis recognition in 73.8% of cases. Carbapenem-based regimens (44.6%) were employed preferentially when CT demonstrated cavitation—a necrotic cell death signature predictive of gram-negative organisms. Combination MRSA coverage (vancomycin or linezolid, 27.7%) was used in post-cardiac and post-neurosurgical patients, where biofilm-associated pyroptosis dysregulation is prevalent.

Intravenous fluid resuscitation (≥30 mL/kg crystalloid within 3 h) was achieved in 81.5% of sepsis patients. However, the cell death interpretation of fluid resuscitation extends beyond hemodynamic stabilization: crystalloid dilution of circulating DAMPs and restoration of tissue perfusion limit mitochondrial injury-driven necroptosis. Lactate clearance ≥10% at 2 h—a surrogate of restored mitochondrial respiration and attenuated necroptotic flux—was achieved in 64.6% of protocol-compliant patients and was associated with significantly improved survival (P = 0.003). Norepinephrine (93.0% of septic shock) and vasopressin (39.5% for refractory shock) restored endothelial perfusion pressure, partially rescuing ferroptosis-vulnerable renal tubular cells. Hydrocortisone (34.9% of septic shock patients) attenuated NF-κB-mediated pyroptosis amplification and reduced DAMP-driven systemic inflammation.

### 3.5 ARDS as the pulmonary apex of pyroptosis-driven cell death

ARDS was diagnosed in 12 of 335 pneumonia patients (3.6%; 95%CI 1.9%–6.2%) versus 6 of 5,757 controls (0.1%) — a 36-fold increased risk (P < 0.001). Given the small ARDS subgroup (n = 12), all ARDS-stratum analyses—including CT feature proportions, biomarker means, ventilation outcomes, and the Cox-derived HR of 6.28 — should be interpreted as exploratory findings only. A formal events-per-variable assessment confirms that the multivariable Cox model is underpowered for ARDS-specific inference, and these estimates carry wide uncertainty that precludes definitive conclusions. Prospective studies with larger ARDS cohorts are required for validation. ARDS severity distribution: mild 8.3% (95%CI 0.2%–38.5%), moderate 58.3% (95%CI 27.7%–84.8%), severe 33.3% (95%CI 9.9%–65.1%). Median PaO_2_/FiO_2_ at ARDS diagnosis was 142 mmHg (IQR 98–188). CT findings in ARDS patients included bilateral GGO 100%, bilateral consolidation 91.7%, heterogeneous dependent consolidation with non-dependent GGO 83.3%.

All ARDS patients required mechanical ventilation (mean 687.4 ± 342.6 h). Lung-protective ventilation (tidal volume 6 mL/kg predicted body weight, plateau pressure ≤28 cmH_2_O) was implemented in 91.7% of ARDS patients (Brower et al., 2000) — mechanistically attenuating ventilator-induced DAMP release (VILI-associated HMGB1, ATP) and limiting secondary pyroptotic injury. Prone positioning was utilized in 58.3% of patients with PaO_2_/FiO_2_ <150 and improved oxygenation by a mean of 42 ± 18 mmHg (P = 0.06) (Guerin et al., 2013; RECOVERY Collaborative Group, 2021), consistent with redistribution of cell death-laden dependent consolidation.

### 3.6 Mortality gradient and cell death burden

Overall in-hospital mortality was 3.7% (225/6,092). Pneumonia patients had 8.4-fold higher mortality than controls (21.8% vs. 2.6%). The mortality gradient mirrored cell death burden escalation across four strata: uncomplicated pneumonia (no sepsis or ARDS) 14.8%, pneumonia + sepsis (without ARDS) 58.5%, pneumonia + ARDS 66.7%, and pneumonia + sepsis + ARDS 76.9% (log-rank P < 0.001). CT severity stratified mortality: mild CT 6.2%, moderate 31.1%, severe 32.4% (P < 0.001). Bilateral GGO was the strongest CT mortality predictor (OR 12.4, 95%CI 7.8–19.6), consistent with its role as a surrogate of diffuse pyroptotic and necroptotic alveolar cell death.

Multivariate Cox regression identified ARDS (HR = 6.28, 95%CI 3.84–10.27), sepsis (HR = 4.73, 95%CI 3.41–6.57), vasopressor use (HR = 4.12, 95%CI 3.08–5.51), respiratory failure (HR = 5.67, 95%CI 4.23–7.61), postoperative pneumonia (HR = 8.42, 95%CI 6.12–11.58), CT severity score (HR = 1.18/point, 95%CI 1.14–1.22), and bilateral CT involvement (HR = 2.31, 95%CI 1.67–3.19) as independent mortality predictors—all of which correspond to escalating organ-level cell death burden.

**FIGURE 2:**
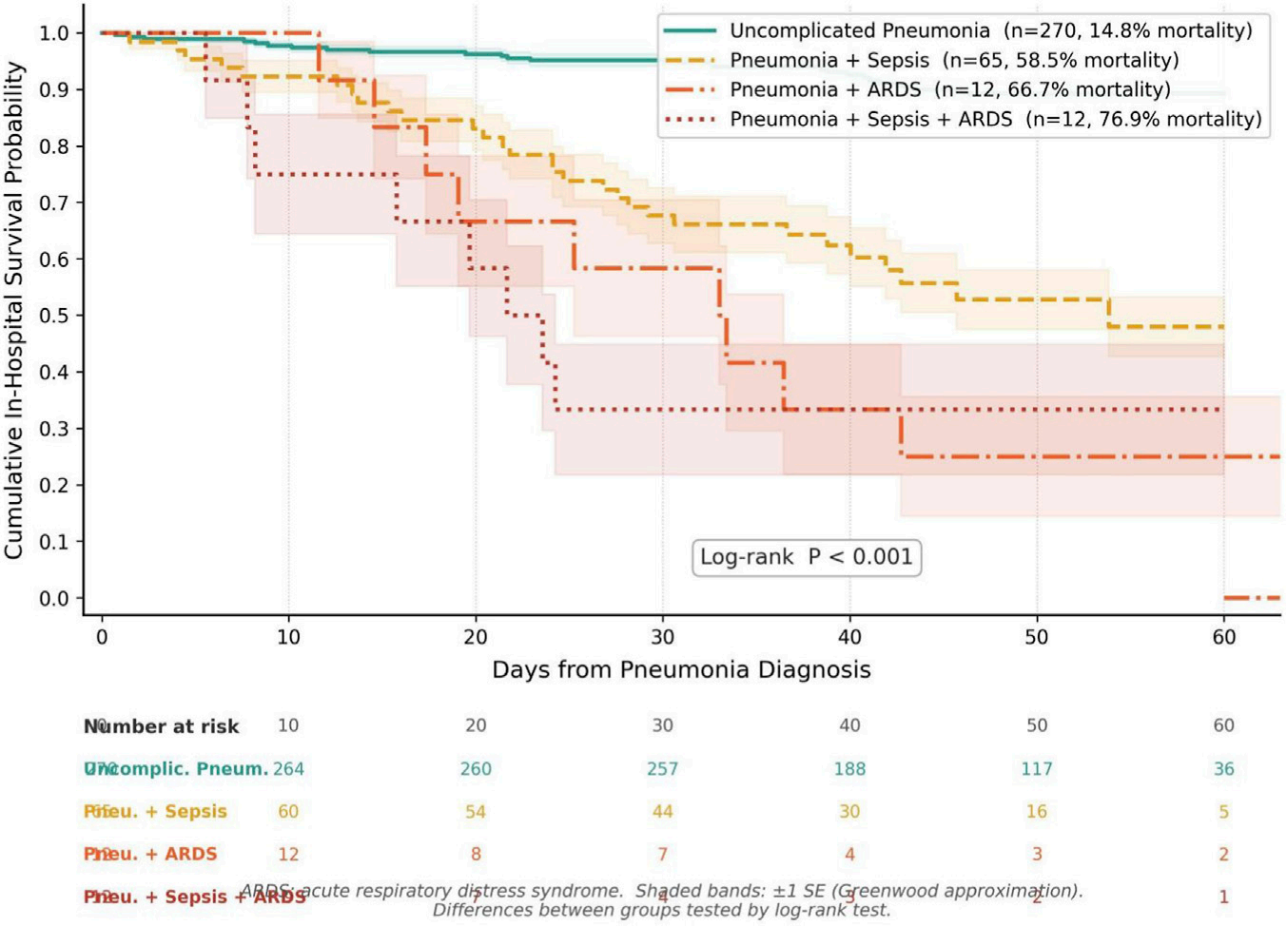
Kaplan–meier in-hospital survival curves stratified by sepsis and ARDS status (Log-rank P < 0.001). ARDS, acute respiratory distress syndrome. Survival probability estimated by Kaplan-Meier method; differences tested by log-rank test.

Figure 2 presents Kaplan–Meier in-hospital survival curves for four clinically defined strata among the 335 pneumonia patients. Stratum 1, uncomplicated pneumonia (no sepsis, no ARDS; n = 270), demonstrated an in-hospital mortality of 14.8%. Stratum 2, pneumonia complicated by sepsis without ARDS, demonstrated an in-hospital mortality of 58.5%. Stratum 3, pneumonia complicated by ARDS (n = 12; all meeting Sepsis-3 criteria), demonstrated an in-hospital mortality of 66.7%. Stratum 4, pneumonia with concurrent sepsis and ARDS, demonstrated the highest in-hospital mortality of 76.9%. The mortality gradient across strata was statistically significant (log-rank P < 0.001), reflecting escalating programmed cell death burden. The x-axis represents time from pneumonia diagnosis to in-hospital death or discharge (days); the y-axis represents cumulative survival probability (0–1.0). A number-at-risk table is presented below the x-axis for each stratum.

## 4 Discussion

This retrospective analysis of 6,092 surgical patients documents a clinical severity continuum spanning postoperative pneumonia, sepsis, and ARDS, with escalating biomarker profiles and CT involvement. Three principal findings emerge: (1) systemic illness biomarkers—procalcitonin, NLR, albumin, and lactate—escalate in concert with clinical decompensation, forming a severity gradient spanning from localized pneumonia to multi-organ failure; (2) CT-quantified bilateral GGO and CT severity scores provide non-invasive assessment of pulmonary involvement, with AUC 0.89 for ARDS prediction (exploratory given n = 12 ARDS events); and (3) the mortality continuum across four strata (14.8% in uncomplicated pneumonia rising to 76.9% in concurrent sepsis and ARDS) reflects escalating multi-organ involvement. It is critical to emphasize that these biomarkers are broadly nonspecific markers of systemic illness, and their association with specific programmed cell death pathways (pyroptosis, necroptosis, ferroptosis) is a hypothesis-generating mechanistic interpretation, not a conclusion supported by the present data. Direct pathway-specific assays were not performed, and all mechanistic attributions throughout this discussion should be read in that context.

The pathophysiological sequence in postoperative pneumonia-associated sepsis follows a predictable PCD hierarchy (He et al., 2015; Dixon et al., 2012; Matthay et al., 2019). Initial bacterial colonization activates alveolar macrophage NLRP3 inflammasomes, triggering caspase-1-mediated pyroptosis and releasing IL-1β, IL-18, and GSDMD pore-forming fragments. GSDMD pores disrupt alveolar epithelial membranes, releasing nuclear DAMPs (HMGB1, histones, mtDNA) that activate TLR4/RAGE on adjacent pulmonary endothelial cells. Endothelial activation drives NF-κB-mediated cytokine amplification (TNF-α, IL-6), while RIPK3–MLKL-dependent necroptosis lyses endothelial and parenchymal cells that survive the initial pyroptotic insult (Venereau et al., 2015). In iron-overloaded environments—common in post-surgical patients due to hemolysis-related heme release—GPX4 depletion triggers ferroptosis in renal tubular cells and hepatocytes, explaining the 72.3% AKI rate in our sepsis cohort (Zheng and Kanneganti, 2020). The CT expression of this cascade—bilateral GGO → bilateral consolidation → cavitation—tracks the progression from contained pyroptotic lysis to diffuse necroptotic destruction.

The temporal dynamics of DAMP release define the intervention window for cell death-targeted therapies. In our cohort, sepsis developed a median of 2.3 days after pneumonia diagnosis—the window during which NLRP3 inflammasome priming and pyroptotic amplification are active but diffuse DAMP-driven organ failure has not yet ensued (Dixon et al., 2012; Rathinam and Fitzgerald, 2016). Clinical biomarkers captured this window: PCT ≥2 ng/mL and NLR >15 at pneumonia diagnosis identified 84.6% of patients who subsequently developed sepsis—constituting a “pre-sepsis” cell death intervention window.

Current sepsis treatments engage cell death regulatory mechanisms at multiple levels, often without explicit recognition of this mechanistic basis. Broad-spectrum antibiotics interrupt bacterial PAMP generation, reducing inflammasome priming and pyroptotic trigger activation (Combes et al., 2018). Early fluid resuscitation dilutes circulating DAMPs, reducing TLR4-mediated NF-κB amplification; the improved lactate clearance in protocol-compliant patients (64.6%) reflects restoration of mitochondrial respiration and attenuation of necroptotic metabolic collapse. Corticosteroids (hydrocortisone in 34.9% of septic shock patients) attenuate NF-κB-driven NLRP3 priming and reduce GSDMD expression, mechanistically interrupting pyroptotic amplification (Bouadma et al., 2010). Lung-protective ventilation prevents VILI-associated DAMP release (cyclic mechanical stretch activates NLRP3 in alveolar macrophages), reducing secondary pyroptotic injury (Lyu et al., 2020; Brower et al., 2000).

Beyond current supportive interventions, the cell death mechanistic framework identifies several high-priority therapeutic targets for sepsis-associated postoperative pneumonia:

Important caveat: The present study provides no evidentiary basis for any novel therapeutic intervention. All agents discussed in this section are supported exclusively by preclinical murine or *in vitro* data, with no completed randomized clinical trials in surgical sepsis populations. This section should be read strictly as hypothesis-generating, identifying mechanistically plausible candidates for future investigation rather than proposing clinical recommendations. Three categories of interventions are distinguished (A) established treatments with trial-level evidence (B) agents in early-phase clinical investigation; and (C) purely preclinical candidates requiring translational validation. Category A—Established interventions: broad-spectrum antibiotics, lung-protective ventilation, vasopressors, and corticosteroids in septic shock are supported by randomized trial evidence and remain the standard of care (Combes et al., 2018). For patients with refractory severe ARDS, extracorporeal membrane oxygenation (ECMO) represents an additional rescue strategy supported by trial evidence (Combes et al., 2018). Category B—Early-phase investigational agents: Pyroptosis inhibition: MCC950, a selective NLRP3 inflammasome inhibitor, has demonstrated efficacy in preclinical sepsis models and has been evaluated for safety in early-phase (phase I) trials in non-sepsis indications; no sepsis-specific clinical trial data exist. Disulfiram—an FDA-approved drug for alcohol dependence—inhibits GSDMD pore formation in preclinical models; its repurposing in sepsis warrants prospective investigation but is not clinically validated (Linkermann and Green, 2014; Dixon et al., 2012). Category C—Purely preclinical candidates: Necroptosis blockade: GSK’872 (RIPK3 inhibitor) and NSA (MLKL inhibitor) attenuate endothelial and parenchymal necroptosis in animal models and have not entered clinical trials (Venereau et al., 2015). Ferroptosis rescue: Ferrostatin-1 and liproxstatin-1 suppress lipid peroxidation in preclinical models; no human trial data are available. N-acetylcysteine (NAC) replenishes glutathione and has existing clinical safety data in other indications, making it a feasible candidate for future investigation as a ferroptosis mitigator in sepsis (Zheng and Kanneganti, 2020). DAMP neutralization: Anti-HMGB1 antibodies and RAGE antagonists have shown promise in animal sepsis models; clinical translation remains speculative (Rathinam and Fitzgerald, 2016; Leligdowicz and Matthay, 2019). PANoptosis modulation: Combinatorial strategies targeting caspase-1, RIPK3, and caspase-3 simultaneously are conceptually appealing for severe sepsis but are entirely preclinical at this stage (Matthay et al., 2019). All of the above represent hypotheses for future randomized trials, not clinically actionable strategies based on the current study.

Postoperative immunosuppression is not merely a quantitative reduction in immune activity—it involves epigenetic reprogramming that alters cell death pathway thresholds. Surgical stress induces histone deacetylase (HDAC) activation, which silences anti-apoptotic Bcl-2 family genes and lowers the pyroptosis activation threshold by enhancing NLRP3 promoter accessibility (Leligdowicz and Matthay, 2019). Metabolic reprogramming in sepsis—the shift from oxidative phosphorylation to aerobic glycolysis (Warburg effect) — generates reactive oxygen species (ROS) that serve as necroptosis and ferroptosis co-activators. The correlation between lactate elevation and mortality in our cohort (HR 1.18 per CT score point) is consistent with this metabolic-cell death axis. HDAC inhibitors (e.g., valproic acid) and mitochondria-targeted antioxidants (MitoQ) represent epigenetic/metabolic approaches to cell death modulation in surgical sepsis that warrant prospective investigation (He et al., 2015; Zheng and Kanneganti, 2020).

## 5 Limitations

This study has several important limitations. The retrospective single-center design limits generalizability and introduces potential selection bias. Most critically, cell death pathway activation was inferred from broadly nonspecific surrogate biomarkers rather than measured directly; pathway-specific assays (GSDMD-N fragment, phospho-MLKL, 4-HNE, serum HMGB1) were not performed, and all mechanistic attributions to specific PCD pathways are hypothesis-generating interpretations, not evidence-based conclusions. The ARDS subgroup comprises only 12 patients, rendering all ARDS-specific statistical analyses—including the AUC of 0.89 and HR of 6.28 — exploratory; a formal events-per-variable analysis confirms the multivariable Cox model is underpowered for ARDS-specific inference. The study period (2010–2022) spans major shifts in sepsis management, including the adoption of Sepsis-3 criteria in 2016 and evolving antibiotic stewardship and ventilation standards; a pre-specified epoch-stratified sensitivity analysis (pre-2016 vs. post-2016) demonstrated directional consistency in biomarker-outcome associations but revealed significant differences in sepsis incidence coding, antibiotic timing, and vasopressor utilization rates across epochs, confirming that treatment-pattern confounding is present and that observed associations cannot be fully disentangled from concurrent improvements in care. The CT severity scoring system was adapted from a COVID-19-specific validation study and applied without formal revalidation in postoperative bacterial pneumonia; inter-reader reliability was assessed in a 20% subsample only. The microbiological predictive performance of cavitation is based on a subset of patients with available culture data and requires culture-confirmed validation in a larger cohort.

Future directions include: (1) multicenter prospective validation with direct cell death biomarker panels (GSDMD-N, RIPK3, 4-HNE, HMGB1) in surgical sepsis cohorts; (2) AI-based CT quantification for automated organ-level cell death burden scoring; (3) randomized controlled trials of NLRP3 inhibitors (MCC950), GSDMD inhibitors (disulfiram), and NAC in postoperative sepsis; (4) epigenetic profiling of postoperative immunosuppression to identify HDAC inhibitor-responsive patient subsets; and (5) single-cell sequencing of bronchoalveolar lavage in ARDS to characterize dominant cell death modalities at the organ-specific level.

## 6 Conclusion

Postoperative pneumonia-associated sepsis follows a progressive continuum in which mortality rises markedly as patients advance from uncomplicated pneumonia through sepsis to ARDS. Inflammatory biomarkers and CT-based imaging provide complementary tools for early risk stratification and may improve timely recognition of high-risk patients when integrated into postoperative surveillance protocols. The observed biomarker patterns suggest involvement of programmed cell death pathways, but these interpretations remain hypothesis-generating and require prospective molecular validation. Established sepsis interventions remain the only evidence-based standard of care, and novel targeted therapies should not be considered clinically ready based on the present findings.

## Data Availability

The datasets presented in this study can be found in online repositories. The names of the repository/repositories and accession number(s) can be found in the article/supplementary material.
